# Attenuation of Histone Methyltransferase KRYPTONITE-mediated transcriptional gene silencing by Geminivirus

**DOI:** 10.1038/srep16476

**Published:** 2015-11-25

**Authors:** Yan-Wei Sun, Chuan-Sia Tee, Yong-Huan Ma, Gang Wang, Xiang-Mei Yao, Jian Ye

**Affiliations:** 1 State Key Laboratory of Plant Genomics, Institute of Microbiology, Chinese Academy of Sciences, Beijing 100101, China; 2 Temasek Life Sciences Laboratory, National University of Singapore, Singapore 117604, Singapore

## Abstract

Although histone H3K9 methylation has been intensively studied in animals and a model plant *Arabidopsis thaliana*, little is known about the evolution of the histone methyltransferase and its roles in plant biotic stress response. Here we identified a *Nicotiana benthamiana* homolog of H3K9 histone methyltransferase KRYPTONITE (NbKYP) and demonstrated its fundamental roles on methylation of plant and virus, beside of leading to the suppression of endogenous gene expression and virus replication. *NbKYP* and another gene encoding DNA methyltransferase *CHROMOMETHYLTRANSFERASE 3* (*NbCMT3-1*) were further identified as the key components of maintenance of transcriptional gene silencing, a DNA methylation involved anti-virus machinery. All three types of DNA methylations (asymmetric CHH and symmetric CHG/CG) were severely affected in *NbKYP-*silenced plants, but only severe reduction of CHG methylation found in *NbCMT3-1-*silenced plants. Attesting to the importance of plant histone H3K9 methylation immunity to virus, the virulence of geminiviruses requires virus-encoded trans-activator AC2 which inhibits the expression of *KYP* via activation of an EAR-motif-containing transcription repressor *RAV2* (*RELATED TO ABI3 and VP1*). The reduction of *KYP* was correlated with virulence of various similar geminiviruses. These findings provide a novel mechanism of how virus trans-activates a plant endogenous anti-silencing machinery to gain high virulence.

RNA silencing is a highly conserved pathway that is involved in diverse biological processes. It is particularly important to plants to defense against invasive nucleic acid such as infectious pathogens and endogenous transponsable elements (TEs)^1^. RNA silencing is initiated with the processing of double-stranded RNA (dsRNA) into 21–24 nucleotides (nt) small interfering RNAs (siRNAs) or other types of regulatory small RNA (sRNA) to target invasive nucleic acid. The function of siRNA is to silence viral genome (mainly 21-nt) or other invasive nucleic acid such as TEs (mainly 24-nt) through Post-transcriptional gene silencing (PTGS) or Transcriptional gene silencing (TGS), respectively^2^,^3^. To protect genome integrity the majority of TEs has to be in a transcriptionally silenced state that is epigenetically propagated from generation to generation. Transcriptionally active TEs may also trigger RNA silencing to degrade the mRNA. The mechanism of controlling propagating of TEs and infectious pathogens may be closely related. In addition to RNA silencing, many organisms, especially plants, have developed other sophisticated epigenetic mechanisms, e.g. chromatin-mediated regulation, to control propagation of molecular parasites within its genome^2^,^3^. On the other hand, it is interesting to note that TEs are evolutionarily capable of reversing silencing. However the mechanism is not yet understood.

It has become evident that chromatin structure and dynamics during all biological regulation processes in eukaryotic organisms are increasingly important for antiviral therapy and the regulation of viral gene expression^4^,^5^,^6^. Chromatin structure changes such as histone modification, nucleosome location, and DNA methylation play a central role in controlling of the virus life cycle and the transformation of a normal cell to a cancer cell. Some viruses organize their genomes into chromatin-like structure such as minichromosomes, which undergoes different histone modifications to facilitate complicate functions in virus life cycles including replication. For example, the genomes of Simian virus 40 (SV40), Hepatitis B virus (HBV) and Herpes Virus form minichromosomes that enable them to replicate in the nucleus^4^,^5^,^6^. Chromatin structure and dynamics have a wide role in plant developmental and cellular processes. However less work has been done to understand the relationship between modification of chromatin and pathogen (including viruses) resistance in plant. Thus, understanding the mechanisms employed by viruses to modulate chromatin function would have broader implications to our understanding in the control of viral diseases in general^7^.

The control of both endogenous and exogenous invasive nucleic acid is established by forming heterochromatin which largely depends on epigenetic modifications on histone and chromatin structure^3^,^7^. DNA methylation acts as a repressive marker in transcription. In the model plant *Arabidopsis thaliana*, *de novo* methylation of any cytosines in CG, CHG, and CHH (H = A, T, or C) is initiated by DOMAINS REARRANGED METHYLTRANSFERASE 1 (DRM1) and DRM2. *De novo* DNA methylation patterns may then be maintained during DNA replication in a siRNA-independent manner^8^. CHG methylation maintenance involves the coordinated action of CHROMOMETHYLTRANSFERASE 3 (CMT3) and several SET domain H3K9 histone methyltransferases, whereas DNA METHYLTRANSFERASE 1 (MET1) maintains CG methylation patterns^9^,^10^,^11^. In *A. thaliana*, histone H3K9 dimethylation (H3K9me2) and CHG methylation are reciprocally maintained through a self-reinforcing loop between DNA methyltransferase CMT3 and H3K9 methyltransferase KRYPTONITE (KYP, an ortholog of human EHMT2 (also termed as G9a)^12^,^13^. Furthermore H3K9me2 is enriched in heterochromatic regions and is an important repressive mark required for the silencing of TEs^2^,^3^. To date, it is not clear that whether these two epigenetic DNA methylation and histone methylation have any one of those precursor of each other during the transcriptional process. Recently, *CMT* genes were identified from various plant species by using high-throughput sequencing and genomics analysis^14^. However no KYP homologs has been functional identified from other plants yet. Further investigation on the genetic diversity of KYP/CMTs pair and their unique function is essential for a better understanding in KYP/CMT-mediated epigenetic regulation in plants. It is also of great importance to understand the biological significance of the interdependent methylations on DNA and histone conferred by this pair of epigenetic effectors.

Epigenetic modification can exert TGS via RNA-dependent DNA methylation (RdDM). The process of DNA methylation is mediated by RNA, which is produced by RNA polymerase IV. To perform *de novo* methylation, RNA polymerse IV recognizes the target site and synthesizes single-stranded RNA transcript. The RNA transcript is then converted to double-stranded RNA. Ultimately, the siRNA produced is loaded to effector Argonaute 1 (AGO1) or AGO4 to guide PTGS or TGS complex to the target DNA. DNA methyltransferase DRM2 exists in complex with the siRNA effector AGO4 and preferentially methylates one DNA strand *de novo*, which likely acts as the template for RNA polymerase V-mediated noncoding RNA transcripts. This siRNA-dependent and strand-biased DNA methylation is also positively correlated with strand-biased siRNA accumulation^8^.

Geminiviruses cause increasingly serious threats to economic crops such as cotton, cassava, tomato, Jatropha and so on^15^,^16^,^17^,^18^. Geminiviruses possess single-stranded circular DNA in their monopartite or bipartite genome and have a coding capacity of 6–7 proteins. Monopartite geminiviruses contain only one genomic component which is named as DNA-A. Many monopartite begomoviruses are associated with a single protein-encoding betasatellite. On the other hand, bipartite geminiviruses contain separate DNA-A and DNA-B in their genome. These viruses replicate in the nucleus with a rolling circle mechanism via a replicative intermediate. The replicative intermediate is in dsDNA form and is associated with histones to form minichromosomes^19^. Similar to host chromatin, geminiviral minichromosomes are subjected to epigenetic modification which potentially causes TGS^20^. To counter this, geminiviruses encode proteins to block TGS^19^. In addition to activation of late viral genes, the multifunctional AC2s from some of geminiviruses such as *African cassava mosaic virus* and *Indian cassava mosaic virus* have been found to be a suppressor of PTGS^21^, while AC2 from a same host cassava geminivirus *Sri Lankan cassava mosaic* cannot suppress PTGS^22^. In contrast, no suppressor of TGS has been identified from cassava geminivirus so far. Further study suggested that *Cabbage leaf curl virus* (CaLCuV) AC2 induces WERNER-LIKE EXONUCLEASE 1 encoded by host, of which the gene locus is transcriptionally silenced due to its repetitive characteristic and has been proposed to be a negative regulator of PTGS^23^. Moreover, C2/L2 of monopartite curtoviruses and betasatellite βC1 protein of *Tomato yellow leaf curl China virus* (TYLCCNV) compromise S-adenosyl methionine methyl cycle^24^,^25^,^26^. Similar to DNA methylation, histone methylation was also proposed as a defense strategy against DNA viruses^20^. Apart from virus-encoded silencing suppressor, plant also encodes endogenous silencing suppressors which allow plant to attenuate or turn off silencing machinery^27^,^28^. However, how chromatin modifications on virus genome affect pathogenesis of geminivirus and how viruses counter or even hijack this system are still unclear. The epigenetic modification on histone for the pathogen-host interaction pair is poorly understood.

In the study, we identified the key histone methyltransferase responsible for histone H3K9 methylation and its roles in plant-geminivirus interaction in *Nicotiana benthamiana*. We first functionally characterized the KYP homolog from *N. benthamiana*. We then took advantage of two newly isolated *Indian cassava mosaic virus* strains, ICMV-Dha and ICMV-SG, which show 95% nucleotide identity but exhibit large difference in virulence^17^,^18^. Phylogenetic analysis showed that ICMV-SG evolved after ICMV-Dha. Our data showed that the transactivator protein AC2 positively regulate the expression of *RAV2*, a transcriptional repressor and an endogenous silencing suppressor. *RAV2* transcriptionally inhibited host histone methyltransferase gene *KYP*. This repression of *KYP* dampened TGS and facilitated virus survival in host. Our results revealed a novel strategy of how virus escapes from host TGS surveillance.

## Results

### H3K9 methylation is essential for DNA methylation and maintenance of TGS in *N. benthamiana*

**Figure 1:**
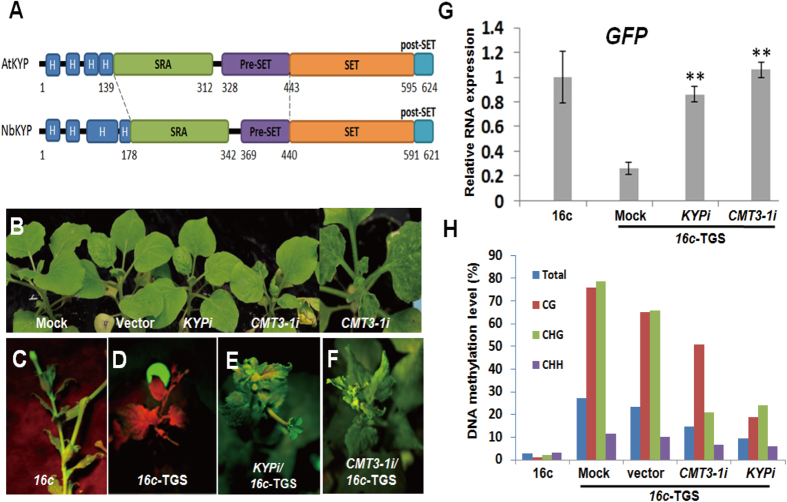
*KYP* and *CMT3-1* are essential for transcriptional gene silencing in *N. benthamiana*. (**A**) Color-coded domain architecture of full-length AtKYP and NbKYP. The letter H indicates α-helix and the number below the domains indicate start and end amino acid. (**B**) Phenotypes of *KYP-* and *CMT3-1-*silenced *N. benthamiana* (*KYPi* and *CMT3-1i*). (**C**). *CaMV35S:GFP* transgenic *N. benthamiana* line *16c*. (**D**) Transgeneration heritable transcriptional gene silencing (TGS) induced by *sTRV:35S* VIGS on progenies of *N. benthamiana16c*. (**E,F**). *16c*-*35S* TGS reversed on *KYP-* and *CMT3-1-*silenced plants (*KYPi* and *CMT3-1i*). (**G**) Relative expression level of *GFP* on the TGS plants, in which respective gene was silenced as indicated as x-axis. Asterisks indicate significant differences for *GFP* expression level between the mock and the indicated silenced plants. (*P < 0.05; **P < 0.01; Student’s *t-*test). (**H**) The percentage of methylated cytosine sites in *35S* promoter region of *35S:GFP N. benthamiana* with indicated genetic background.

Most histone methyltransferases contain a catalytic SET domain. KRYPTONITE (KYP) directly methylates Histone H3 Lysine 9 (H3K9) in *Arabidopsis thaliana*. It contains four predicated N-terminal α-helical segments and two other domains: SRA domain and pre-SET/SET/post-SET domain (Fig. 1A). KYP is phylogenetically distal from other SUVh paralogs in many plant species, including *N. benthamiana* (Supplementary Figure S1A). The KYP family proteins have separated from SUVh superfamily proteins since 500 million years ago when the basal landplant moss *Physcomitrella patens* separated from higher plants. It seems that KYP proteins have fundamental but different function from other SUVh family proteins. The major difference between NbKYP and AtKYP were identified in two domains: the SRA domain and the pre-SET domain. Both the SRA and pre-SET domain of NbKYP are shorter than the corresponding domains of AtKYP, suggesting functional divergence for two homologs (Fig. 1A). The SRA domain is found to be important for recognition of methylated DNA, while pre-SET domain contains positively charged amino acids to mediate the plausible interaction with histone contained nucleosome^13^. The H3K9 methylation mark in *Arabidopsis* is controlled through a self-reinforcing loop between KYP and DNA methyltransferase CMT3^29^. We identified three putative CHROMOMETHYLASE 3 (CMT3) paralogs in *N. benthamiana* based on the draft genome sequence (Supplementary Figure S1B). Among the three paralogs, NbCMT3-1 and NbCMT3-2 are highly similar, with 93% identity, but distal from NbCMT3-3. The expression level of *NbCMT3-1* is the highest among three paralog genes. Next, we next silenced the expression of either *KYP* or *CMT3-1* to functionally analyze their roles in plant developmental regulation by synthetic Tobacco Rattle Virus (sTRV) mediated virus-induced gene silencing (VIGS)^30^, *NbAGO1-1* which has been shown to affect plant development was used as a control^31^. After VIGS, the expression of all these 3 genes was significantly reduced by 70%–80% in each VIGS plants (Supplementary Figure S2). Plant growth was not affected in *KYP*-silenced plants (*KYPi*). In contrast, obvious phenotypes were observed in *CMT3-1*-silenced plants (*CMT3-1i*) as well as *AGO1-1-*silenced plants (*AGO1-1i*) as reported before (Fig. 1B–F). *CMT3-1i* plants presented leave curling symptom whereas *AGO1-1i* plants developed downward leave curling and stunted leaves (Fig. 1B).

To further investigate whether *NbKYP*, *NbCMT3-1* and *NbAGO1-1* are involved in transcriptional gene silencing (TGS) in *N. benthamiana*, we employed the *16c*-TGS system^26^,^32^. *16c*-TGS plants are derived from *GFP*-overexpression 1*6c* line which is generated by transformation of *CaMV 35S*::*GFP* (Fig. 1C). To generate *16c*-TGS plants, TGS of *GFP* was induced by VIGS that targets *CaMV 35S* promoter. As a consequence, *CaMV 35S* promoter is hypermethylated and transcriptionally silenced. This silencing effect is inheritable to the next generations as indicated by GFP fluorescence intensity, *GFP* mRNA level and DNA methylation level in *35S* promoter region (Fig. 1D,G,H). Under UV illumination, GFP was detected in *16c* plant in which *GFP* highly expressed (Fig. 1C). As expected, GFP was not detected in mock- or vector-inoculated *16c*-TGS plant (Fig. 1H). Knockdown of *KYP* or *CMT3-1* resulted in strong *GFP* expression in *16c*-TGS plants, suggesting that TGS failed to be maintained in the absence of *KYP* and *CMT3-1* (Fig. 1E,F). Consistent with the result above, quantitative RT-PCR (qRT-PCR) analysis revealed that *KYP*- and *CMT3-1*-silenced plants expressed comparable level of *GFP* to *16c* line (Fig. 1G). To determine whether re-activation of *GFP* was caused by the loss of DNA methylation *per se*, we analyzed DNA methylation level at the *35S* promoter region of *35S:GFP* within plant genome. The loss of DNA methylation was well correlated with the expression level of *GFP* (Fig. 1H). This is consistent with results in early reports^26^,^31^. Knockdown of *AGO1-1* slightly decreased methylation level compared to vector control. On the other hand, knockdown of *CMT3-1* or *KYP*, which are interdependent, largely decreased DNA methylation, particularly in CHG context which agrees with their functional roles in *Arabidopsis*. Intriguingly, in addition to CHG, CG methylation was reduced remarkably in *KYP*-silenced plants. We then measured expression of *MET1*, the primary DNA methyltransferase for CG, in *KYP*-silenced plants. Surprisingly, *MET1* was not down-regulated in *KYP*-silenced plants, implicating that *KYP* might regulate CG methylation independently of transcriptional regulation of *MET1* (Supplementary Figure S3).

In summary, *KYP*, *CMT3-1* and *AGO1-1* participate in the control of TGS in *N. benthamiana*. Thus loss of *CMT3-1* or *KYP* causes loss of DNA methylation and TGS dysfunction.

### High pathogenicity of ICMV-SG is correlated with transcriptional repression on *KYP*

To further characterize the roles of histone H3K9 and DNA methylation mediated by *KYP*, *CMT3-1* and *AGO1-1* against virus pathogen, we further challenged *KYP-*, *CMT3*-*1-* and *AGO1-1-*silenced plants with a recently identified bipartite geminivirus *Indian cassava mosaic virus* (ICMV-SG strain). Both virus symptoms and viral titer analysis (Supplementary Figure S4) suggested that all of these three genes are not only important for TGS maintenance but also crucial for plant resistance to geminivirus infection in *N. bethamiana*.

**Figure 2:**
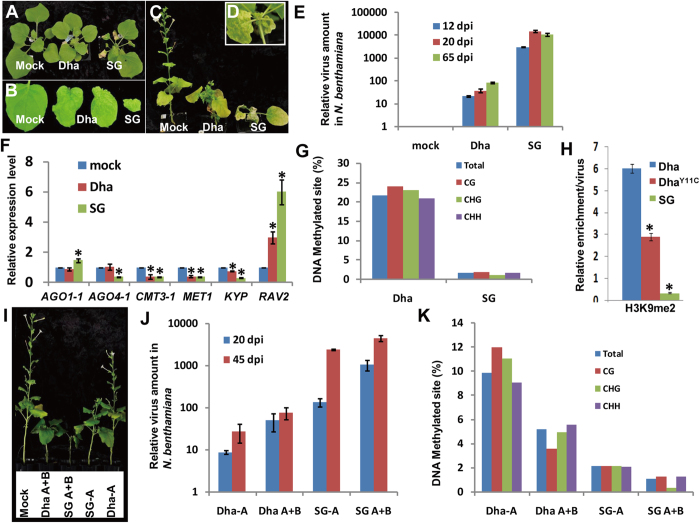
DNA-A of a high pathogenic ICMV strain (ICMV-SG) interferes with plant epigenetic modifications and enhances virus pathogenicity. (**A–D**) Systemic symptoms developed on ICMV-SG- or ICMV-Dha-infected *N. benthamiana.* (**A**) Early infected *N. benthamiana* (12 day post inoculation, dpi) with ICMV-Dha (Dha) and ICMV-SG (DNA-A of SG + DNA-B of Dha) shown typical leaf symptoms. (**B**) Leaves of early stage of infected *N. benthamiana* with mosaic and malformed symptoms (10 dpi). (**C**) Late stage of infected *N. benthamiana* (45 dpi). (**D**) Enlarged view of *N. benthamiana* infected by ICMV-SG. (**E**) The relative virus titer analyzed by quantitative real-time PCR in infected *N. benthamiana* at 12, 20 and 65 dpi. (**F**) Relative gene expression level on plants infected by ICMV-Dha and ICMV-SG, MMA mock buffer inoculation as control at 20 dpi. Asterisks indicate significant differences for the gene expression level between mock and two ICMV strains treated plants at the same time point (*P < 0.05, Student’s *t-*test). (**G**) The percentage of methylated cytosine sites in intergenic region of ICMV-Dha and ICMV-SG at 20 dpi. (**H**) Histone methylation state of ICMV-Dha, ICMV-Dha^Y11C^ and ICMV-SG. Asterisks indicate significant differences between different treatments at the indicated time point (*P < 0.05, Student’s *t-*test). (**I**) Single DNA-A of ICMV-SG (SG-A) infection caused obvious virus symptoms but not ICMV-Dha (Dha-A). (**J**) The relative virus titer in ICMV-SG DNA-A alone infected *N. benthamiana* at 20 and 45 dpi, compared with that of ICMV-SG DNA-A plus ICMV-Dha DNA-B, ICMV-Dha DNA-A, and ICMV-Dha DNA-A plus DNA-B. (**K**) The percentage of methylated cytosine sites in IR of ICMV-Dha and ICMV-SG infected *N. benthamiana* at 45 dpi.

We recently reported that DNA-A of ICMV-SG causes typical geminivirus symptoms^18^. Genome-wide sequence alignment showed that DNA-A of ICMV-SG is 95% identical to that of ICMV-Dha in the whole genome wide. However, ICMV-SG causes more severe symptoms compared to ICMV-Dha. To understand the molecular mechanism of the highly pathogenic DNA-A of ICMV-SG, we did comparative analysis by challenging *N. bethamiana* with the two newly isolated ICMV strains. ICMV-Dha (DNA-A and DNA-B of Dha strain) and ICMV-SG (DNA-A of SG plus DNA-B of Dha strain) were inoculated onto *N. bethamiana* (Fig. 2A,D). In the experiments, the growth of ICMV-Dha- and ICMV-SG-inoculated plants was slightly retarded compared to mock-inoculated plant (Fig. 2A). In addition, the leaves of both of the infected plants displayed severe stunt and downward curl (Fig. 2B,D). Prolonged infection resulted in shortened internodes, dwarfing and high lethality (Fig. 2C). Quantitative PCR (qPCR) has been widely used as a method to quantify geminivirus in our and other groups^33^,^34^. This method was adopted to quantify virus titer in this study. Virus titer in the ICMV-SG infected plant was 150 folds higher than that of ICMV-Dha on 12 days post-inoculation (dpi). The virus amount peaked on 20 dpi and reached 400 folds difference in ICMV-SG compared to ICMV-Dha but went down to 130 folds in a later stage (65 dpi, Fig. 2E). To understand the possible relationship between the virulence and the expression level of host resistance genes such as Salicylic acid (SA) pathway *(Pathogenesis Related* genes, *PRs*) and RNA silencing pathway, the expression of their important components was quantified. Expression level of these two antiviral pathway genes in virus-infected plants was not affected (*AGO1-1* in Fig. 2F and data not shown). Next, we analyzed the expression of plant epigenetic modification pathway genes. Interestingly, these two ICMVs repressed the expression of *KYP* and two DNA methyltransferase genes *MET1* and *CMT3-1.* Noteworthy, the reduction level of *MET1* and *CMT3-1* by the two ICMV strains was similar, whereas the reduction of *KYP* expression was positively correlated with the virulence of two ICMVs (Fig. 2F).

To determine the consequence of down-regulation of the host methyltransferase on virus resistance, we looked into DNA methylation level at the viral Intergenic Region (IR) which shares high sequence similarity between two components of the ICMV (DNA-A and DNA-B). IR contains promoter for viral genes and also serves as the origin of genomic DNA replication. All types of methylations including CG, CHG and CHH in IR region were intensively inhibited by ICMV-SG compared to ICMV-Dha (Fig. 2G). This is consistent with the downregulation of the two DNA methyltransferase genes encoded for the *N.benthamiana* homologs of *AtCMT3* and *AtMET1* (Fig. 2F). As expected, DNA methylation level of IR in ICMV-SG was greatly reduced (1.7%) compared with that of ICMV-Dha (21.7%) (Fig. 2G and details in Supplementary Figure S5A andS5B). To quantify the histone methylation level of viral chromatin, chromatin immunoprecipitation was performed and the result was normalized with virus amount. Expectedly, H3K9me2 level of each DNA-A viral chromatin was much reduced in the IR region, as a consequence of reduced host *KYP* expression in geminivirus infected cells (Fig. 2H).

We have found that DNA-A of bipartite ICMV-SG alone can cause virus symptoms in *N. benthamiana*^18^ (Fig. 2I), suggesting a potential key virulence factor embedded in DNA-A of ICMV-SG. We inoculated DNA-A either from ICMV-SG (SG-A) or ICMV-Dha (Dha-A) on *N. benthamiana*. The viral titer in SG-A alone treated plant was around 100 folds higher than that of Dha-A alone and was almost half the virus titer of the plants that were co-inoculated with DNA-A and DNA-B on 45dpi (Fig. 2J). Similarly, SG-A effectively inhibited DNA methylation whereas Dha-A only weakly interfered with host DNA methylation (Fig. 2K and details in Supplementary Figure S5C and S5D). In summary, high pathogenicity of ICMV-SG is correlated with hypomethylation of viral genome. More important SG-A is sufficient to elicit pathogenesis via inhibition of *KYP* expression and DNA methylation.

### Gain-of-function mutant of AC2 is sufficient to increase pathogenicity of ICMV-Dha

**Figure 3:**
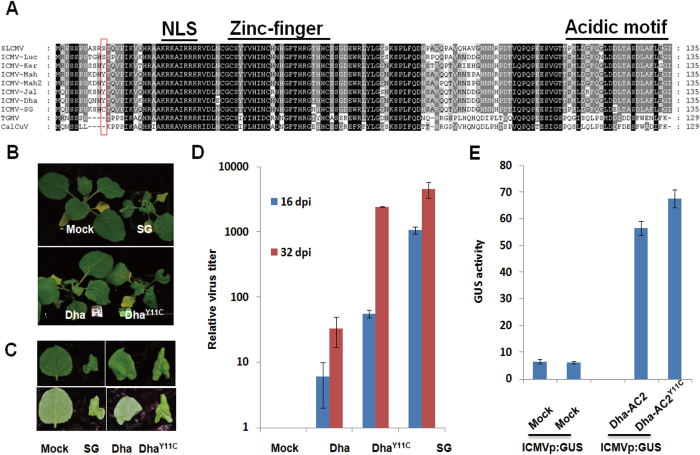
The AC2 protein of ICMV-SG is essential for virulence and transactivation activity. (**A**) Sequence alignment of ICMV-SG AC2 proteins and other cassava infected related mosaic geminiviruses. The position of function domains are indicated top of the figure. NLS: nuclear location signal. The red frame indicated single amino acid difference. (**B,C**) Symptoms of *N. benthamiana* infiltrated with mock, ICMV-Dha, ICMV-SG, and ICMV-Dha^Y11C^. (**D**) Virus titers of ICMV-Dha, ICMV-Dha^Y11C^ and ICMV-SG. (**E**) GUS activity assay result of *N. benthamiana* co-inoculated with AC2s and *GUS* reporter strain. Asterisk indicate significant difference for transactivation activity between Dha-AC2 and Dha-AC2^Y11C^ treated *N. benthamiana* at the same time point (*P < 0.05, Student’s *t-*test).

The high infectivity of DNA-A of ICMV-SG strain prompted us to speculate that ICMV-SG acquires its high pathogenicity through mutation on the protein encoded by DNA-A. Previous studies demonstrated that DNA-A of geminiviruses encodes transcription activator protein (TrAP) which is capable of suppressing PTGS in addition to transactivation of the later stage gene(s) embedded in DNA-B^15^. Therefore, the superior infectivity of ICMV-SG led us to investigate AC2, the TrAP encoded in ICMV-SG. We analyzed amino acid sequence of AC2 from different ICMV isolates and compared their sequence similarity. There are 3 known functional domains or motifs in AC2 proteins, nuclear location signal (NLS), Cysteine-rich Zinc-finger domain which confers DNA-binding activity and the most COOH-terminal acidic motif for the transactivation activity (Fig. 3A). Among the 7 amino acid difference in AC2s of ICMV-SG and ICMV-Dha, an amino acid substitution at the 11^th^ position where tyrosine (Y) is replaced by cysteine (C) in ICMV-SG was noteworthy as these nearby cysteines are shown to be important for homo-dimerization of AC2 which confers its DNA-binding and transactivation activity^23^,^35^ (Fig. 3A). To gain more insight into the substitution, we converted Y11 to C11 in ICMV-Dha (denoted as Dha^Y11C^). Strikingly, Dha^Y11C^ gained virulence as indicated by the symptoms presentation such as dwarfing in the respective plants (Fig. 3B). A closer observation revealed that the gain-of-function mutation Dha^Y11C^ causes the hosts to develop severe stunted and downward-curled leaves, which have been observed in ICMV-SG infected plants (Fig. 3C). Consistent with the severity of symptoms, the virus amount of Dha^Y11C^ accumulated in plants was 9-fold and 6-fold more than Dha on 16 and 32 dpi respectively (Fig. 3D). Nonetheless, the virus amount in the Dha^Y11C^infected plant was much less than that of SG, indicating that other amino acids of AC2 protein or DNA-A proteins of ICMV-SG are indispensable for its virulence. A single point mutation of Dha^Y11C^ cannot fully explain the high virulence of ICMV-SG.

As AC2 is a transactivator protein, we further analyzed the transactivation activity for Dha-AC2 and the gain-of-function mutant Dha-AC2^Y11C^. We found that Dha-AC2^Y11C^ has stronger transactivation activity than that of Dha-AC2 (Fig. 3E). In summary, our findings suggested that single amino acid substitution at the 11^th^ position (Y→C) of AC2 was sufficient for partial gain-of-virulence and transactivation activity in ICMV.

### Gain-of-function mutant of AC2 strongly enhances the TGS inhibition

RAV is a plant transcription factor that is required for the regulation of RNA silencing and is upregulated by virus silencing suppressor of potyvirus HC-Pro and carmovirus P38 in Arabidopsis^36^. A recent report suggested that *Arabidopsis* RAV represents a control point that can be readily subverted by viruses to antagonize antiviral mechanism such as RNA silencing^36^. We identified two RAV parologs from *N. benthamiana* genome (Supplementary Figure S6). The expression of a putative transcriptional repressor gene *NbRAV2*, which encodes a putative endogenous silencing suppressor, was further found to be positively correlated with the virulence of geminiviruses and negatively correlated the gene expression of *NbKYP* (Fig. 2F). This suggested that KYP might be a molecular target of geminviruses. This result also implied a possible causal relationship between *KYP* and *RAV2*.

**Figure 4:**
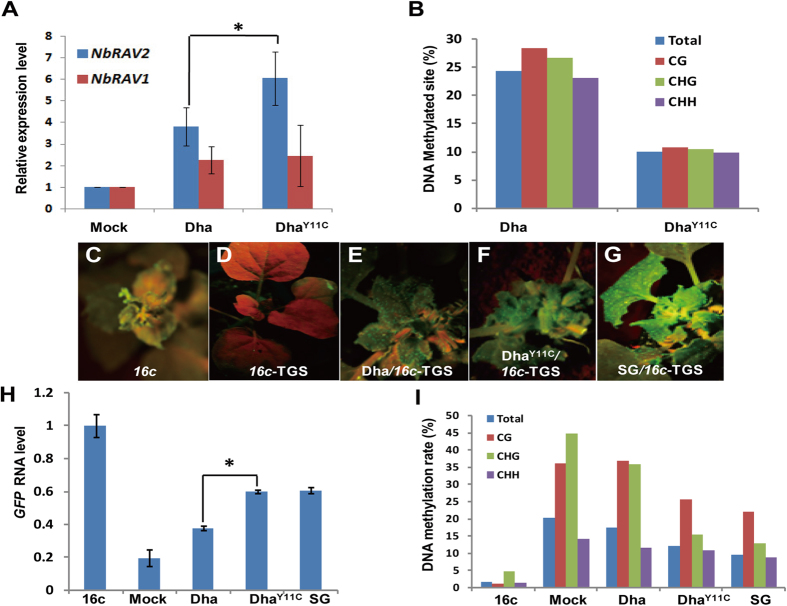
Gain-of-function mutant of AC2 strongly enhances the TGS inhibition. (**A**) Relative expression level of *RAV1* and *RAV2* in ICMV-Dha and ICMV-Dha^Y11C^ infected *N. benthamiana*. Asterisk indicates significant difference for *NbRAV2* expression level between ICMV-Dha and ICMV-Dha^Y11C^ infected plants at the same time point (*P < 0.05, Student’s *t-*test). (**B**) The percentage of methylated cytosine sites in common region of ICMV-Dha and ICMV-Dha^Y11C^ infected *N. benthamiana* at 20 dpi. (**C**) *N. benthamiana16c* line (**D–G**) Phenotypes of mock (**D**), ICMV-Dha (**E**), ICMV-Dha^Y11C^ (**F**) and ICMV-SG (**G**) infected plants and partial inhibition of TGS in *35S:GFP* silenced *N. benthamiana.* (**H**) Relative expression level of *GFP* on the ICMV strains infected plants. Asterisk indicates significant difference for *GFP* expression level between ICMV-Dha and ICMV-Dha^Y11C^ infected plants at the same time point (*P < 0.05, Student’s *t-*test). (**I**) The percentage of methylated cytosine sites in *35S* promoter region of *35S:GFP N. benthamiana* infected by ICMV-Dha, ICMV-Dha^Y11C^ and ICMV-SG.

Thus, we tested *RAV* expression level in Dha and Dha^Y11C^ infected plants. *RAV2* expression in Dha and Dha^Y11C^ infected plant is 4 times and 6 times higher than mock, respectively (Fig. 4A). However, *RAV1* expression was found to be similar in both of Dha infected and Dha^Y11C^ infected *N. benthamiana.* DNA methylation analysis showed that the level of 3 types of DNA methylation in Dha^Y11C^ was reduced by half compared to Dha (Fig. 4B and details in Supplementary Figure S5E and S5F). We also analyzed H3K9 methylation level. It was found that Dha^Y11C^ partially inhibited H3K9 methylation compared to Dha (Fig. 2H).

The inhibition of *KYP* and loss of repressive epigenetic marks are associated with high pathogenicity of SG-A. To investigate whether the loss of repressive epigenetic marks could result in block of TGS, we employed *16c*-TGS line in the experiment. Under UV illumination, *16c* produced intense green fluorescence (Fig. 4C). On the other hand, *16c*-TGS line inoculated with mock did not produce green fluorescence under UV illumination (Fig. 4D). We then inoculated Dha, Dha^Y11C^ and SG onto *16c*-TGS line. All 3 types of AC2 were capable of re-activating GFP expression but the ability of induction was different. Dha weakly activated GFP expression as indicated by dimmed fluorescence (Fig. 4E), whereas SG elicited strong fluorescence (Fig. 4G). Notably, fluorescence intensity induced by Dha^Y11C^ was higher than Dha but lower than SG, suggesting that inducing ability of Dha^Y11C^ was partially enhanced (Fig. 4F). Quantitative PCR analysis confirmed the observation, of which GFP expression level induced by Dha^Y11C^ is as high as that by SG (60% of *16c* line) (Fig. 4H). In contrast, Dha induced low level of *GFP* (40% of *16c* line) but the level was higher than mock. To determine whether re-activation of GFP expression by inhibition of TGS, was attributed to loss of methylation *per se*, we analyzed methylation status at *GFP* promoter region. Consistently, *GFP* expression was negatively correlated with methylation level (Fig. 4I). Dha slightly reduced methylation whereas Dha^Y11C^ and SG intensively inhibited methylation. Noteworthy, both of CHG and CG methylation were significantly reduced in the cases of Dha^Y11C^ and SG, whereby only CHG methylation was reduced in the case of Dha (Fig. 4I). This implied that the gain-of function AC2 mutant targets a host factor that has great impact on CG and CHG methylation to control TGS. Our result showed that KYP may cause CG and CHG methylation, therefore KYP may potentially be one of the host factors by geminivirus. In summary, we found that single point mutant AC2^Y11C^ strongly inhibits TGS by reducing DNA methylation.

### *RAV* negatively regulates plant resistance to geminivirus

**Figure 5:**
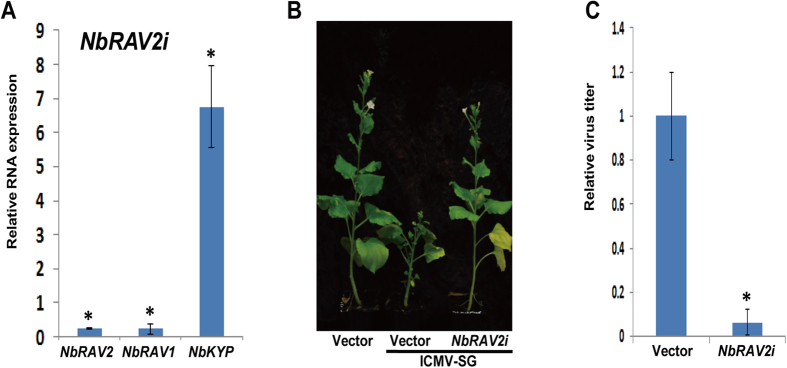
*RAV2* negatively regulates the resistance of *N. benthamiana* to geminivirus. (**A**) Relative expression level of *RAV* in *sTRV:NbRAV2* inoculated *N. benthamiana*. Asterisk indicates significant difference for gene expresstion level between housing gene and respective genes as indicated as x-axis at the same time point (*P < 0.05, Student’s *t-*test). (**B**) Symptoms of ICMV-SG in *sTRV:NbRAV2* inoculated *N. benthamiana*. (**C**) Relative ICMV-SG virus titer in *sTRV:NbRAV2* inoculated *N. benthamiana*. Asterisk indicates significant difference for viral titer between mock and *RAV2-*silenced plants at the same time point (*P < 0.05, Student’s *t-*test).

To identify the relationship between *RAV* and *KYP*, we knocked down the expression of *RAV* by inoculating *sTRV:NbRAV2* onto *N. benthmiana*. The relative RNA expression of *NbRAV1 and NbRAV2* was reduced to less than 10%. Interestingly, the expression of *NbKYP* was highly upregulated than those of *sTRV* vectors treated *N .benthminiana* (Fig. 5A). To determine the impact of knockdown of *RAV* on geminivirus accumulation, we further challenged *NbRAV2-*silenced *N. benthmiana* with ICMV-SG and analyzed the relative virus titer in these plants. The virus titer in *NbRAV2-*silenced *N. benthmiana* was ten times less than that of the vector control (Fig. 5B,C). In bipartite geminivirus CaLCuV-infected or betasatellite betaC1-overexpressing Arabidopsis, *AtRAV1* and *AtRAV2* were also activated (Supplementary Figure S7), indicating a conserved strategy to counter host resistance to geminiviruses by transactivation of an endogenous transcription repressor. In summary, *RAV* is required for suppression of RNA silencing. Geminiviruses infection induced the expression of a transcription repressor *RAV*, which further represses the expression of TGS gene *KYP* in order to assist virus to counter TGS surveillance.

## Discussion

To our knowledge, this is the first investigation for plant virus on how high virulence evolves by transcriptionally suppression of plant histone modification. Human viral pathogens such as RNA virus Human Immunodeficiency Virus and DNA virus Herpes simplex virus encode regulatory proteins (Tat and VP16), which are essential for efficient transcription of the viral genome and reprogramming the host transcription to facilitate virus replication in host cells^37^,^38^. Plant viral pathogens including DNA virus geminiviruses also encode protein to counter host defense by hijacking host transcription^39^. In this study, we showed that a novel function of the geminivirus protein AC2, a transactivation protein of ICMV, functions as a suppressor of TGS by transcriptionally manipulating host TGS multiple components in *N. benthamiana* to create a favorable environment for virus propagation. To illustrate, *MET1* and *CMT3* is repressed by another geminivirus protein AC1 embedded in DNA-A^40^. The expression of *RAV2* was also repressed by AC2 and was inversely correlated to the expression of *KYP* and its downstream events such as histone methylation and DNA methylation, indicating that a complex transcriptional reprogramming strategy is overtaken by geminiviruses. The reduced capability of methylation allows the ICMV-SG minichromosome to escape from TGS. As shown by others and here, DNA methylation is a basic strategy adopted by geminiviruses to repress gene expression^40^. ICMV-SG may gain high virulence by further repress the expression of *KYP* by activating an endogenous suppressor. Although the evidences of RAV-KYP antagonistic relationship has been shown here, whether the transcription repressor RAV2 directly binds to promoter of *KYP* to interfere its transcription remains unknown. Unlike Dha strain, AC2 of ICMV-SG strain has PTGS suppressor activity. This is similar to AC2 of ICMV-NB1 strain^22^. Although the loss-of-AC2 function experiment demonstrated the essential role of the 11^th^cysteine in PTGS suppression, the gain-of function AC2 mutant failed to gain PTGS suppressor activity (data not shown). This is different from the partial success of gain-of function assay in TGS for AC2 (Fig. 4), suggesting the mechanism of AC2 PTGS suppression may differ from that of TGS.

KYP and MET1 are responsible for CHG and CG methylation in Arabidopsis respectively^9^,^12^. We observed that CG methylation level at *35S:GFP* in *KYP-*silenced- *16c*-TGS line was reduced (Fig. 1L). The same observation was found in the suppression of TGS caused by gain-of function AC2 mutant-Dha^Y11C^ and SG AC2 (Fig. 4G). We speculate that KYP might be involved in CG methylation in *N. benthamiana*, directly or indirectly. Future experiments are required to dissect the detailed mechanism of histone code-modifying enzyme NbKYP on histone and DNA methylation. For instance, it will be useful to target NbKYP to a specific gene locus to provide an insight into KYP-mediated histone and DNA methylation. On the other hand, the homologs of CMT3 but not the KYP family proteins are functionally important in plant development throughout the entire life cycle (both the vegetative and reproductive stages). The discrepancy between the reciprocal KYP and CMT3 pair in developmental process is likely due to the LIKE HETEROCHROMATIN PROTEIN 1 (LHP1) family proteins, which are essential for recruitment of CMT3 to target sites in an evolutionarily conserved manner. LHP1 is one of the crucial components of the POLYCOMB REPRESSIVE COMPLEX1 (PRC1) in plants, and it functions downstream of PRC2 to repress genes expression by modifying both lys27 and lys9 of H3 for orchestrated development^2^,^3^.

It has been well accepted that geminivirus replication occurs by rolling-circle mechanism in which ssDNA is converted to circular dsDNA Replicative Form (RF) (Supplementary Figure S8). In plants, mobile elements and satellite sequences are recognized by RNA-dependent DNA methylation machinery for transcriptional suppression by hypermethylation. Similarly, replicative form of geminiviral genomic DNA is recognized by Pol IV and ssRNA is synthesized. Subsequently, RNA-dependent RNA polymerase 2 (RDR2) converts ssRNA to dsRNA which is then digested by Dicer-like 3 (DCL3) into 21-24nt siRNA duplexes^3^. Next, siRNA is loaded to AGO4 to target the specific sequence via recognition of intergenic transcript produced by Pol V. The assembly of RdDM complex which consists of DRM2 and RDM2 methylates intergenic region^3^. KYP and CMT3 in turn are recruited to the target site and epigenetically modify histone H3 tail and CHG DNA methylation respectively, resulting in epigenetic silencing of virus minichromosome. As a result, viral genome fails to be transcribed and replicated^13^. To achieve successful infection, virus circumvented by this defense mechanism has to develop a strategy to escape from epigenetic silencing^1^. Our findings suggested that ICMV-SG has evolved and acquired the ability to escape as evidenced by its high virulence. The RAV homologs in *N. benthamiana* seem not only to function as a repressor for PTGS but also for TGS. We propose that ICMV-AC2 may interfere TGS by suppressing the expression and/or the activity of KYP by activation of *NbRAV2* which is a putative transcription repressor (Supplementary Figure S8).

The role of histone modification on plant-geminivirus interaction needs further work to understand how the process of each histone tail modification affects geminiviruses. Zhou *et al.* proposed that a plant host factor histone H3 is involved in a bipartite geminiviral DNA complex for intracellular trafficking and cell-to-cell movement via interacting with nuclear shuttle protein and movement protein^41^. As KYP targets H3 histone tail, it is possible that KYP may interfere with virus mobility within and between the cells. However, more work has to be done to understand the role of KYP in plant immune response.

KYP was demonstrated to positively regulate long-term defense gene priming which increases the responsiveness of plant immune response^42^. The study suggested that KYP promotes Salicylic acid (SA)-dependent system acquired resistance (SAR) by methylating uncharacterized gene locus encoding suppressor genes of SA^42^,^43^. As SA is one of the major plant defense pathways, KYP appears to be an important regulatory factor in plant long term immune response. Consistently, our results showed that KYP improves host defense against invading virus by hypermethylation of minichromosome. Together with the induction of SAR, it is thought that KYP may be a critical cellular factor that controls the propagation of genomic DNA or replicative intermediate in dsDNA form^43^. Hence, our study showed that mutation on AC2 harbored by ICMV caused disastrous symptoms in plant. Previous reports have shown that SAR can be inherited to the next generation by RNA-directed DNA methylation and histone H3 Lysine-9 methylation^43^. In fact, when transgenic plant lines expressed multiple siRNAs species upon ACMV infection, *de novo* DNA methylation and an increased proportion of H3K9 at intergenic region were observed^44^. It was proposed that the transgenerational effect is transmitted by hypomethylation of CG at the locus encoding suppressor gene of SAR genes^43^. However, it is possible that repressive mark can be passed down to the next generation by conserving the restrictive state of chromatin in a semi-conservative manner. KYP contains SRA domain which can be recruited to replication fork during DNA replication. Therefore, daughter DNA strands will carry the identical marks due to the self-reinforcing loop^13^. Nevertheless, the mechanism of how the repressive marks are conserved in chromatin remodeling during gametogenesis remains elusive.

Apart from SA-related immune response, it has also been demonstrated that herbivore-induced jasmonic acid (JA)-mediated defensive genes are also subjected to histone modification^45^. We have demonstrated that geminiviruses have evolved to interfere with plant MYC2-regulated JA resistance to favor vector and virus transmission^33^. In this report, we showed that geminivirus repressed the expression of *KYP* and two DNA methyltransferase genes, *MET1* and *CMT3-1*. This might be another possible strategy to repress plant anti-whitefly JA-mediated immune pathway as *NbCMT3* has been shown to be involved in JA signaling pathway^14^. It will be interesting to identify these KYP regulated gene locus which encodes protein for resistance to whitefly. These KYP regulated locus may play significant roles in viral disease pandemic via the possible effect on whitefly population.

## Methods

### Virus induced gene silencing (VIGS)

For VIGS experiments, partial sequences of *NbAGO1-1, NbCMT3-1*, *NbRAV2* and *NbKYP* coding region were amplified using *Pfu* DNA polymerase (Thermo Scientific) with primers listed in supplementary Table S1. The DNA fragments were cloned into *psTRV2*^30^. Plasmids were introduced into *Agrobacterium tumefaciens* AGL1 strain by electroporation. *N. benthamiana* plants were grown in an insect-free growth chamber at 25 °C under 12 h light/12 h dark cycle. Ten days after VIGS, inoculation of ICMV was performed as described previously^17^.

### TGS suppressor activity assays

*N. benthamiana 35S*-GFP transgeneline *16c* was kindly provided by Dr. David Baulcombe. Transcriptional gene silencing for *35S*-GFP transgene (*16c-*TGS) in *N. benthamiana* was induced by VIGS vector carrying *Cauliflower mosaic virus* (CaMV) *35S* promoter fragment as performed as in previous studies^26^,^32^. *16c-*TGS seeds were germinated and silenced plants were selected by GFP imaging as described before^46^. TGS suppression assays were carried out by silencing individual genes with VIGS followed by GFP imaging using Nikon N90 digital camera (Tokyo, Japan) equipped with UV and yellow filters.

### DNA isolation, bisulfite sequencing, and Chromatin immunoprecipitation

Genomic DNA was extracted from plant leaf samples using DNeasy Plant Mini kit (Qiagen, Valencia, CA). Bisulfite treatment was performed with the EpiTect Bisulfite kit (Qiagen) according to the manufacturer’s instructions. Two micrograms of genomic DNA were subjected to sodium bisulfite conversion. The treated DNA was amplified using Dream Taq DNA polymerase (Fermentas) with gene specific primers and purified using Gel Extraction kit (Qiagen). The PCR products were then cloned into pGEM-T Easy Vector (Promega) and 12–30 individual clones from at least 3 biological samples for each treatment were sequenced. DNA cytosine methylation in the CG, CHG, and CHH context was analyzed and displayed using CyMATE (http://katahdin.mssm.edu/kismeth/revpage.pl). Primers were designed against converted templates and are listed in Supplementary Table S1. Chromatin immunoprecipitation was performed as reported before using ChIP Assay Kit (Millipore, 17–295) and H3K9me2 antibody^33^. Virus infected plants were used for ChIP assay. About 3 g of *N. benthamiana* was harvested and fixed in 1% formaldehyde solution under vacuum for 10 min. Glycine was added to a final concentration of 0.125 M, and the sample was treated with vacuum for an additional 5 min. After three washes with distilled water, samples were frozen in liquid nitrogen. The resulting DNA samples were purified with the QIAquick PCR purification kit (Qiagen). The experiments were repeated with three independent biological samples. The relative abundance of the indicated DNA fragments was normalized using the *N. benthamiana ACTIN* promoter as a control and virus DNA amount were further used for the normalization of relative histone modification level per viral DNA.

### Virus Inoculation and titer analysis

For ICMV infection, *N. benthamiana* plants with 4–6 true leaves were infiltrated with *Agrobacterium* carrying DNA-A and DNA-B from either ICMV-Dha or ICMV-SG as described previously^17^,^18^. Infiltration of *Agrobacterium* containing DNA-A alone was used as a control.

### Quantitative PCR (Q-PCR)

Quantitative RT-PCR (qRT-PCR) was conducted as reported before^47^. Q-PCR was used for viral titration^48^,^49^.

### GUS activity assay

Promoter:*GUS* reporter were constructed by PCR amplification and used for trans-activation assay. Leaves of *N. benthamiana* were agroinfiltrated with the indicated constructs. Two days after infiltration leaves were harvested and frozen in liquid nitrogen. Each treatment was repeated eight times. GUS quantitative assay and histochemistry were performed as described^33^.

## Additional Information

**How to cite this article**: Sun, Y.-W. *et al.* Attenuation of Histone Methyltransferase KRYPTONITE-mediated transcriptional gene silencing by Geminivirus. *Sci. Rep.* **5**, 16476; doi: 10.1038/srep16476 (2015).

## Supplementary Material

Supplementary Information
