# Supplementary material for: Attenuation of Histone Methyltransferase KRYPTONITE-mediated transcriptional gene silencing by Geminivirus
Source: Sci Rep. 2015 Nov 25;5:16476. doi: 10.1038/srep16476 (PMC4658475; doi:10.1038/srep16476)
Supplement: Supplementary Information [file srep16476-s1.pdf]

Additional information

Attenuation of Histone Methyltransferase KRYPTONITE-mediated transcriptional gene silencing by Geminivirus

Yan-Wei Sun [1*], Chuan-Sia Tee [2*], Yong-Huan Ma [1*], Gang Wang [2], Xiang-Mei Yao [1], Jian Ye [1,2#]

[1]State Key Laboratory of Plant Genomics, Institute of Microbiology, Chinese Academy of Sciences, Beijing 100101, China

[2] Temasek Life Sciences Laboratory, National University of Singapore, Singapore 117604, Singapore

*** Equal contribution**

[#] Correspondence and requests for materials should be addressed to jianye@im.ac.cn

Table S1. Primers used in this work

Primer	Sequence (5'-3')	Purpose
NbAGO1-Xb5	ATATATCTAGACAAGCAACAAGAGAGTTGACTTT	VIGS
NbAGO1-BH3	TGTCTGGATCCCAGGATTGGGATTGAAGTTCATT	VIGS
NbCMT3-1-Xb5	ATATATCTAGAGATTGAAGAAGATGACAATGCTT	VIGS
NbCMT3-1-BH3	TTGAATGGGATCCAGATGTGGAGAGA	VIGS
NbKYP-BH3	TGTGTGGATCCAATATCCTGGCAATGAAA	VIGS
NbKYP-Xb5	ATATATCTAGATGTATTCCCAATTCCATTTC	VIGS
NbRAV1-Xb5	AATAATCTAGATAGTAGCCAAAGTTATGTGTTGAC	VIGS
NbRAV1-BH3	TCTGTGGATCCACATCACCTAAGTTAGCTCACTTTT	VIGS
NbEF1a-F	TGGTGTCCTCAAGCCTGGTATGGTTG	Q-PCR
NbEF1a-R	ACGCTTGAGATCCTTAACCGCAACATTCTT	Q-PCR
NbAGO4-F	AGTGGCCGTCAATATCTC	Q-PCR
NbAGO4-R	TAATGTGCTCAGGCTTCC	Q-PCR
NbAGO1-1-F	ATGGGAGAGAGCTAGATCTTTTGA	Q-PCR
NbAGO1-1-R	CAAGGGGAATTCGTCTAGAGAGC	Q-PCR
NbCMT3-1-F	TATGAGCTTTGTTGGTGGCAGTT	Q-PCR
NbCMT3-1-R	TGGCCCTGTCAATTTATAGTAAT	Q-PCR
NbKYP-F	ACCGCACTTCTCAGAAAGGA	Q-PCR
NbKYP-R	GCCAACAGAGACTGCATCAA	Q-PCR
NbMET1-F	CCAGGAGCTGACTGGCGTGACCT	Q-PCR
NbMET1-R	GGGTCAGTAATGGAAGTAGGGAA	Q-PCR
NbRAV1-F	TTCAAGTTCAGGTTCAAGCTC	Q-PCR
NbRAV1-R	ACCACTGCAACTAGTCATACTAT	Q-PCR
NbRAV2-F	TTGATTTTAAGGCGAGAAATTT	Q-PCR
NbRAV2-R	ACCACTGCAACAAGTCATATT	Q-PCR
DNA-A-qF	CTGCACAATGTGGGACCCTTTG	Q-PCR
DNA-A-qR	CTTCGCCCTAATGACAGAGACC	Q-PCR
AtTUB2-F	GGTTGAGCCTTACAACGCTACTCTG	Q-PCR
AtTUB2-R	GACCAGGGAACCTCAGACAGCAA	Q-PCR
AtRAV1-F	AGTAAACGGCGTCGTAATGG	Q-PCR
AtRAV1-R	TTTCTCTGCGTGATGTTTCG	Q-PCR
AtRAV2-F	TTCCTCAGCCTAACGGAAGA	Q-PCR
AtRAV2-R	CAAGAAAAGCTAAATCGCCG	Q-PCR
AC_2-Dha-Y11C-F	GATTGGCACCTGAGTACAGTGGTTCTGTGAGGG	Point mutation
AC_2-Dha-Y11C-R	CCCTCACAGAACCACTGTACTCAGGTGCCAATC	Point mutation
DNA-B-ProF	CACCCGGCCCCGCAGGGGATATGCGTAATAATT	Promoter cloning
DNA-B-ProR	CATGCAACAACCATATGCAATACTCACACAT	Promoter cloning
AC_2-SG-SalI F	AATATGTCGACATGCAACCTTCATCTCCCTC	Protein expression
AC_2-SG-XbaI R	ATTAATCTAGAAATACCCTCAAGAAACGCCAGGT	Protein expression

Figure S1

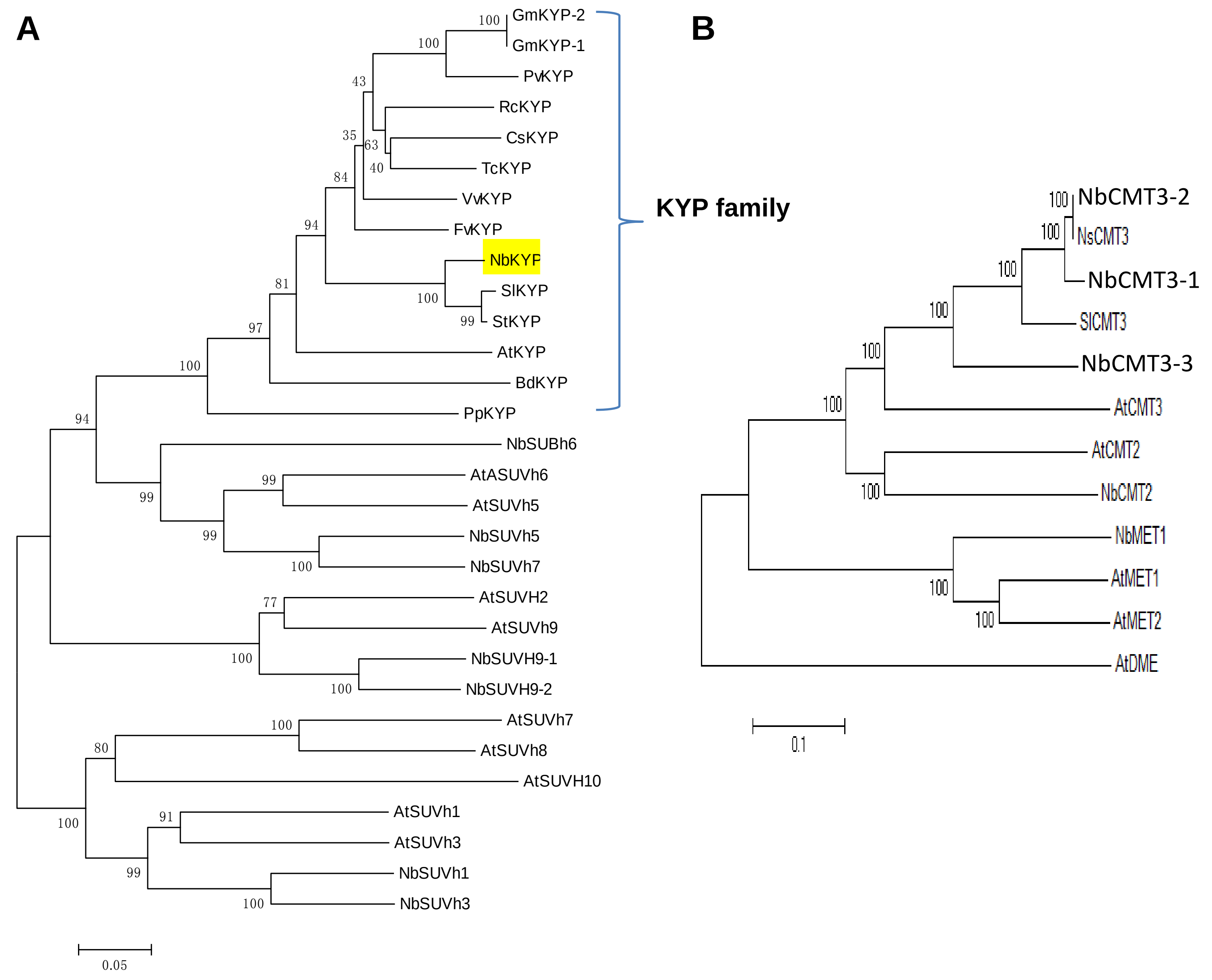

Figure S1. Phylogenetic anlaysis of KYP (A) and CMT3 (B) homologs and paralogs from plants.

Figure S2

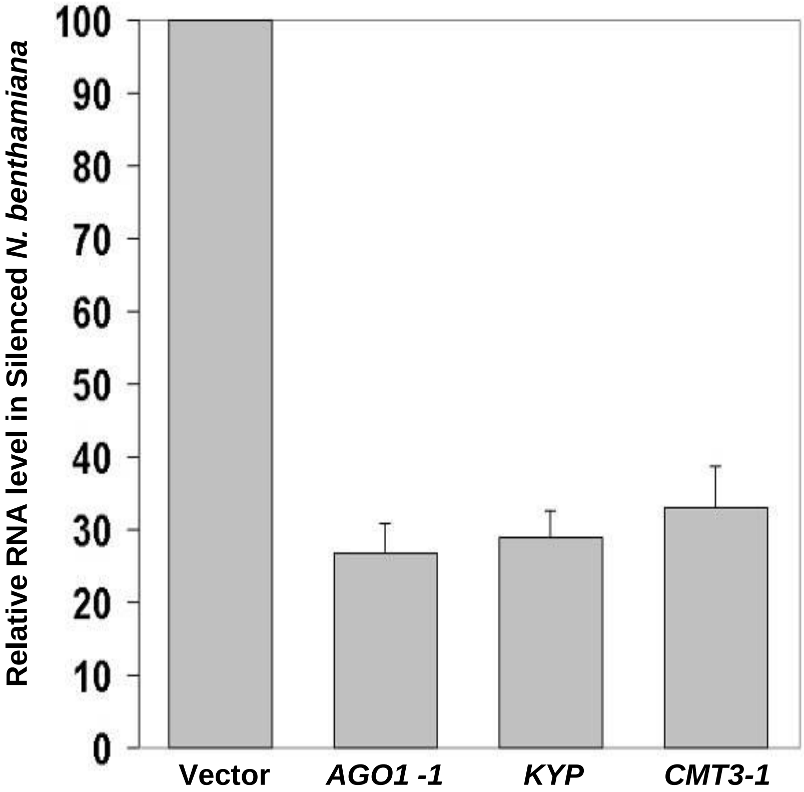

Figure S2. Relative gene expression in *CMT3-1-*, *KYP-*, *AGO1-1-*VIGS plants.

Figure S3

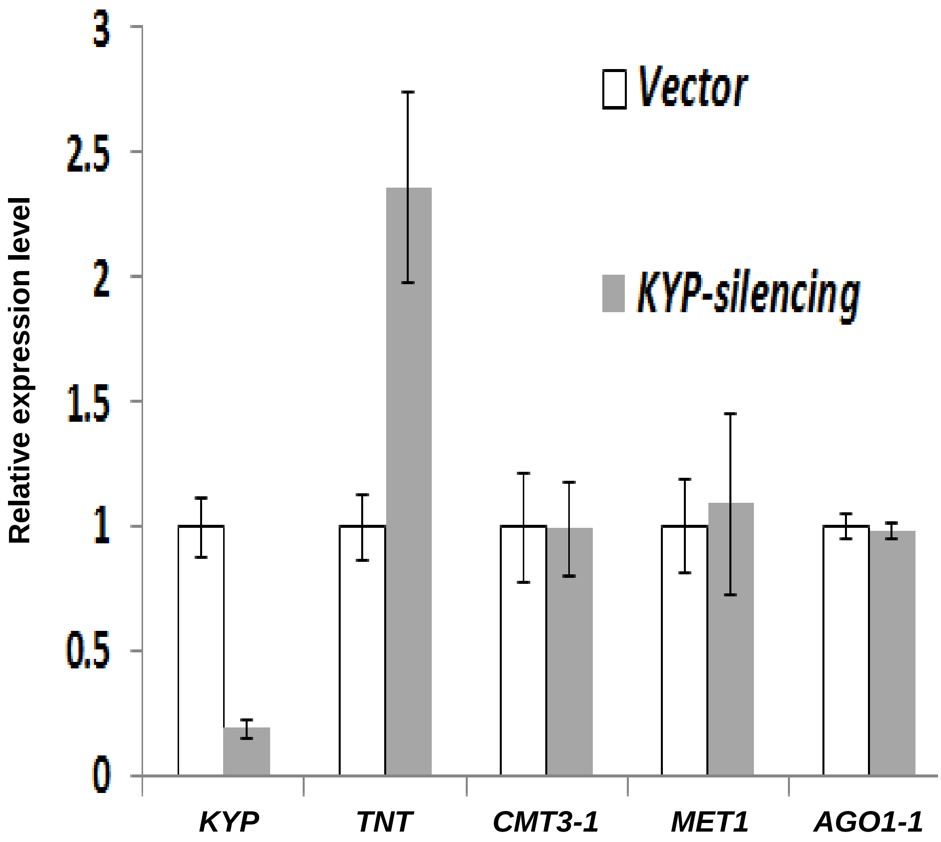

Figure S3. No significant expression change of known genes in DNA methylation in *KYP*-silenced *N.benthamiana.*

Figure S4

A

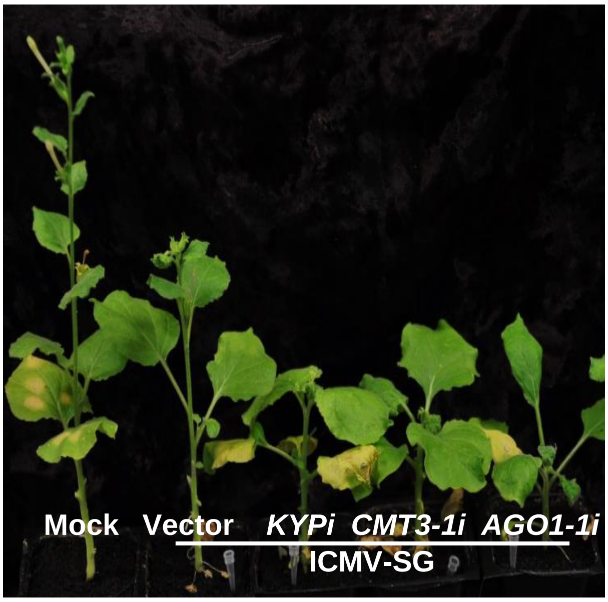

B

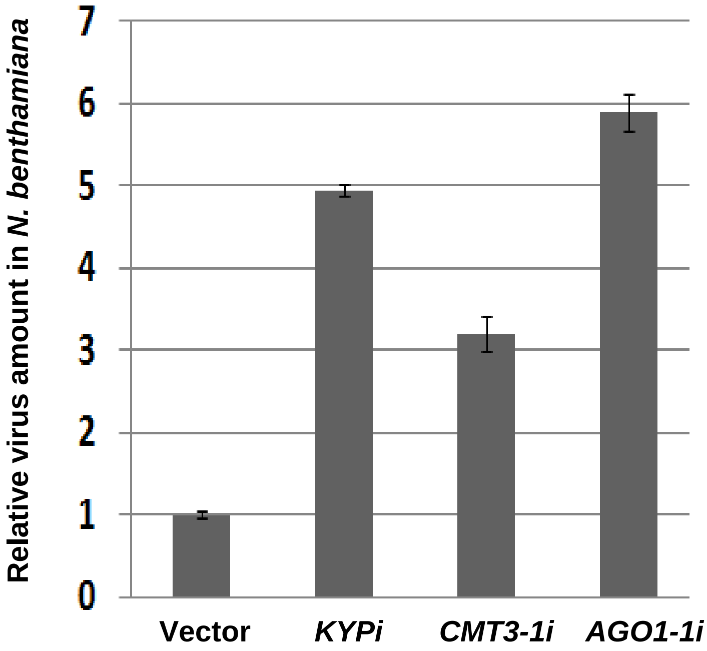

Figure S4. *KYP* and *CMT3-1* are required for geminivirus resistance in *N.benthamiana*.

A. Symptoms of ICMV-SG in *KYP*- or *CMT3-1*-silenced *N. benthmiana*.

B. Relative ICMV-SG virus titer in *KYP*- or *CMT3-1*-silenced *N. benthmiana*.

Figure S5

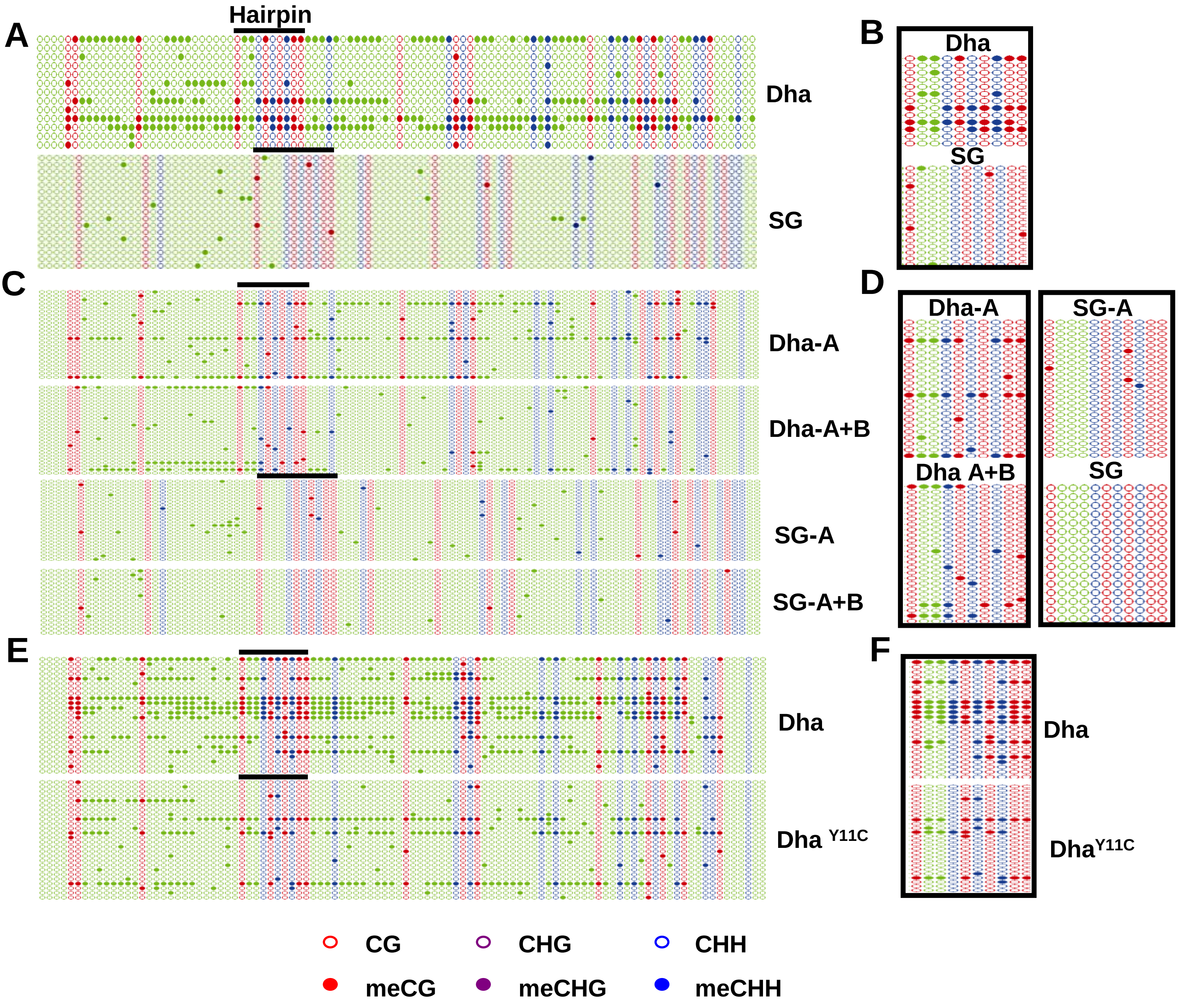

Figure S5. Diagram of DNA methylation patterns in hairpin region of various ICMVs infected *N. benthamiana*.

A. Diagram of DNA methylation patterns in hairpin region of ICMV-Dha and ICMV-SG infected *N. benthamiana*.

B. Highlighted DNA methylation patterns in hairpin region of Figure S5A.

C. Diagram of DNA methylation patterns in hairpin region of ICMV-Dha and ICMV-SG infected *N. benthamiana* either of DNA-A alone or DNA-A plus DNA-B.

D. Highlighted DNA methylation patterns in hairpin region of Figure S5C.

E. Diagram of DNA methylation patterns in hairpin region of ICMV-Dha and ICMV-Dha^{Y11C} infected *N. benthamiana*.

F. Highlighted DNA methylation patterns in hairpin region of Figure S5E.

Figure S6

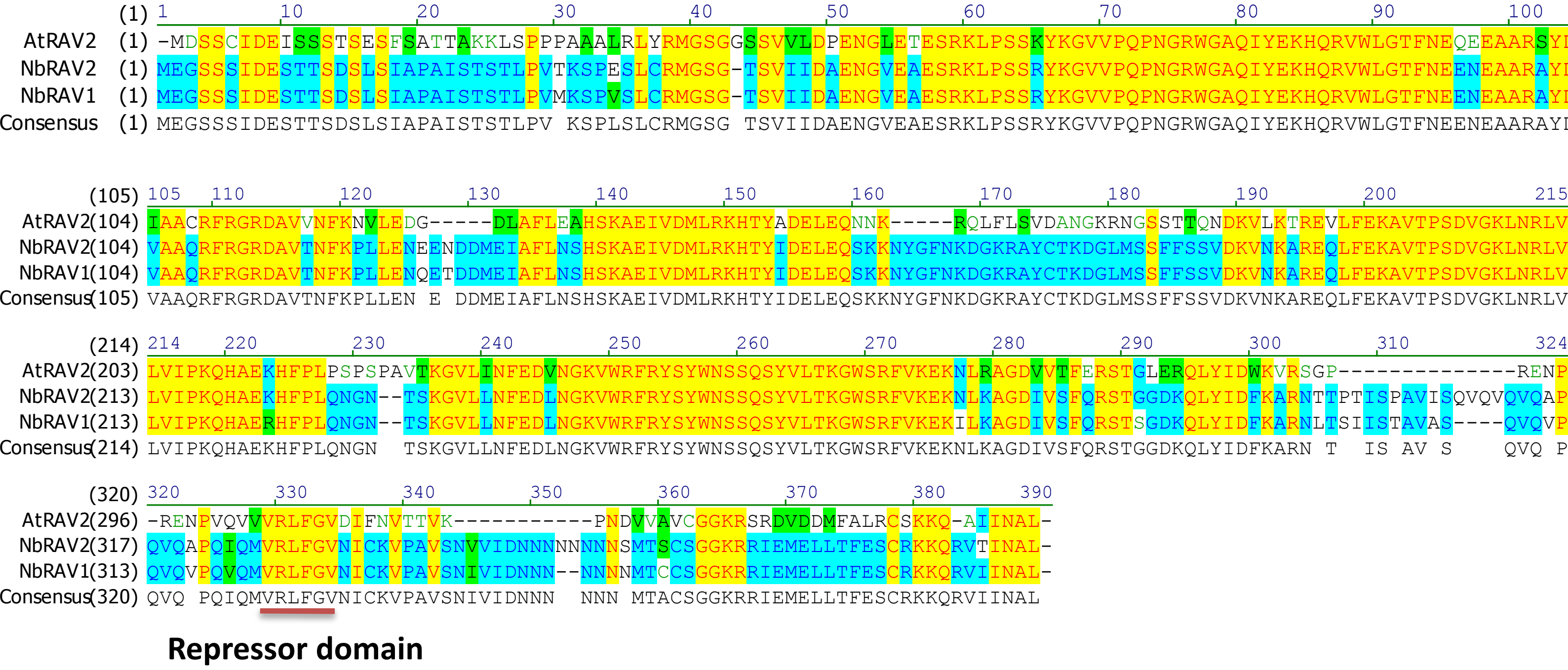

Figure S6. Protein alignment of Arabidopsis RAV2 and two RAV protein paralogs from *N. benthamiana*.

Figure S7

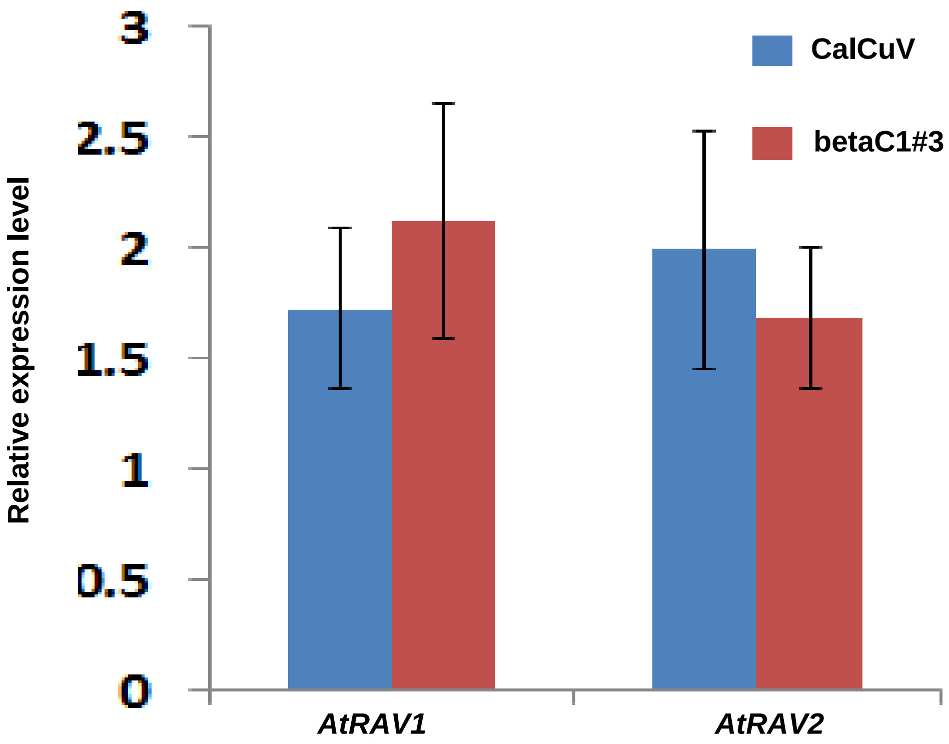

Figure S7. CalCuV infected or betaC1 overexpression Arabidopsis plants accumulate higher *AtRAV1* and *AtRAV2* mRNA level.

Figure. S8

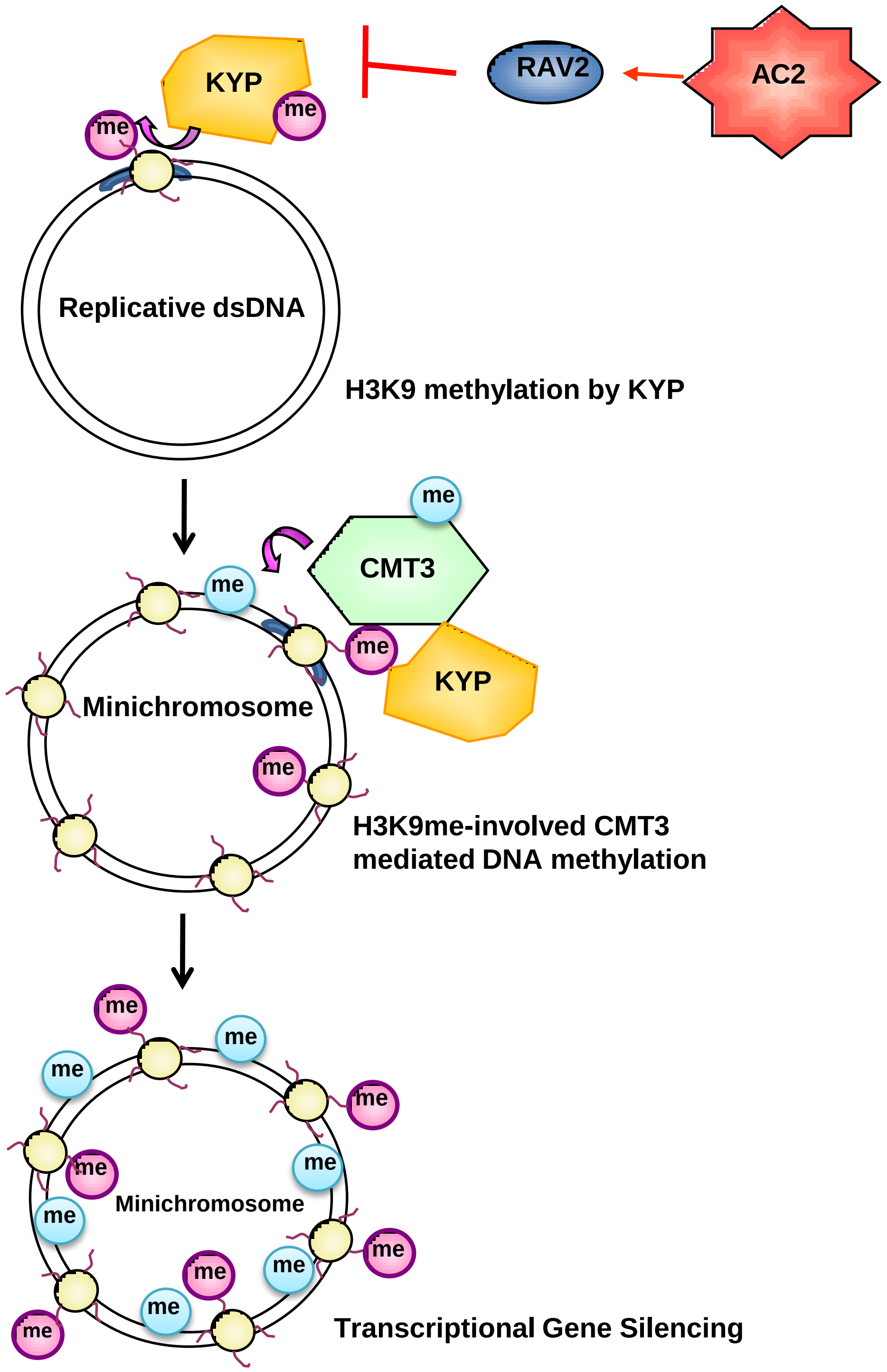

Figure S8. Working model

Histone methyltransferase KYP and DNA methyltransferase CMT3 are recruited to siRNA target site and epigenetically modify H3 tail and viral DNA respectively, resulting in epigenetic silencing. ICMV-AC2 protein interferes transcription gene silencing by suppressing the expression and/or inhibiting the activity of KYP by activation of *NbRAV2* which encodes a putative transcription repressor.
